# Challenges Associated with Pancreas and Kidney Retransplantation—A Retrospective Analysis

**DOI:** 10.3390/jcm10163634

**Published:** 2021-08-17

**Authors:** Nina Pillokeit, Sascha Grzella, Panagiota Zgoura, Timm Westhoff, Richard Viebahn, Peter Schenker

**Affiliations:** 1 Department of Surgery, University Hospital Knappschaftskrankenhaus Bochum, Ruhr-University Bochum, 44892 Bochum, Germany; richard.viebahn@kk-bochum.de; 2 Department of General and Visceral Surgery, Städtisches Klinikum Solingen, 42653 Solingen, Germany; saschagrzella@icloud.com (S.G.); peter.schenker@rub.de (P.S.); 3 Medical Department I, University Hospital Marienhospital Herne, Ruhr-University Bochum, 44625 Herne, Germany; panagiota.zgoura@kk-bochum.de (P.Z.); timm.westhoff@elisabethgruppe.de (T.W.)

**Keywords:** simultaneous pancreas–kidney transplantation, retransplantation, mortality, diabetes type 1

## Abstract

Simultaneous pancreas and kidney transplantation (SPK) is an accepted treatment for diabetic patients with renal failure, and is associated with increased survival and quality of life for recipients. There are only a few publications on the outcomes of simultaneous pancreas–kidney retransplantation (Re-SPK) after previous SPK and the loss of function of both grafts. A total of 55 patients with type 1 diabetes mellitus underwent pancreas retransplantation at our center between January 1994 and March 2021. Twenty-four of these patients underwent Re-SPK after a previous SPK. All 24 operations were technically feasible. Patient survival rate after 3 months, 1 year, and 5 years was 79.2%, 75%, and 66.7%, respectively. The causes of death were septic arterial hemorrhage (*n* = 3), septic multiorgan failure (*n* = 2), and was unknown in one patient. Pancreas and kidney graft function after 3 months, 1 year, and 5 years were 70.8% and 66.7%, 66.7% and 62.5%, and 45.8% and 54.2%, respectively. Relaparotomy was performed in 13 out of 24 (54.2%) patients. The results of our study show that Re-SPK, after previously performed SPK, is a technical and immunological challenge, associated with a significantly increased mortality and complication rate; therefore, the indication for Re-SPK should be very strict. Careful preoperative diagnosis is indispensable.

## 1. Introduction

The first pancreas transplantation was performed at the University of Minnesota in 1966 [1,2]. Initially, there were high complication and mortality rates, and low graft survival rates. However, due to the constant development of immunosuppressive agents and improvement of the surgical technique, 1-year patient and graft survival rates of more than 90% can now be achieved. SPK is currently the treatment of choice for patients with type 1 diabetes mellitus and associated renal insufficiency [1,3].

SPK is the only therapeutic option in which patients can live without insulin and dialysis, in a normoglycemic metabolic situation. Because of the constant normalization of blood sugar after transplantation, progression of late diabetic complications and arteriosclerotic vascular disease can be slowed down and long-term survival, as well as quality of life, are greatly improved [1,2,3,4,5,6,7,8].

Acute or chronic graft rejection, graft pancreatitis, graft thrombosis, anastomotic insufficiency and infections, or recurrence of the underlying disease in patients with type 1 diabetes mellitus are the most common causes of acute or chronic graft failure after SPK [9,10,11,12]. For these patients, there is a possibility of re-admission to the waiting list for retransplantation. This can occur as a simultaneous pancreas–kidney retransplantation (Re-SPK), a pancreas after kidney transplantation (PAK), or an isolated pancreas or kidney retransplantation (Re-PT, Re-KT). In particular, combined pancreas and kidney retransplantation after simultaneous pancreas–kidney transplantation is a technical and immunological challenge. Frequently, these are patients with multiple prior abdominal surgeries, advanced atherosclerosis, high immunization status, and significant comorbidity.

Presently, there are numerous publications on isolated pancreas retransplantation [11,12,13,14,15,16,17,18,19,20,21,22,23,24,25], but only a few with a small number of cases of Re-SPK after previous SPK [26,27,28].

Since 1994, 599 SPK and 55 pancreas retransplantations have been performed at our center. Of these, 30 patients underwent pancreas retransplantation after simultaneous pancreas–kidney transplantation, one underwent pancreas retransplantation after isolated pancreas transplantation, and 24 underwent Re-SPK after previous SPK. In this study, the results obtained after Re-SPK are presented. To the best of our knowledge, this is the largest case series on Re-SPK published to date.

## 2. Materials and Methods

This retrospective study included all patients who underwent Re-SPK from a brain-dead organ donor at the Transplantation Center of Bochum, Germany between January 1994 and March 2021. The analysis comprised patients’ files, laboratory values derived from the electronic databank, and the evaluation of EUROTRANSPLANT donor reports. Preoperative cross-match analysis was negative in all cases.

The aim of this study is to investigate patients’ survival, as well as pancreas and kidney graft survival following Re-SPK. With regard to patient survival, the 90-day, 1- and 5-year survival rates were calculated.

Further objectives were the characteristics of the donors and recipients, perioperative parameters, postoperative complications, length of hospital stay, and occurrence of rejection.

Renal graft failure was defined as patient death with a functioning graft, an allograft nephrectomy, or the need for permanent dialysis or retransplantation. Pancreas graft failure was defined as the need for return to exogenous insulin therapy, removal of the pancreas graft, or patient death. Only histologically confirmed rejection was evaluated and classified according to the BANFF classification.

All patients underwent quadruple immunosuppression comprising induction and triple maintenance therapy. Prednisolone and antithymocyte globulin were administered as induction immunosuppression in almost all patients. Maintenance therapy consisted of low-dose prednisolone, cyclosporine, and azathioprine, whereas in the modern era prednisolone, mycophenolate acid, and a calcineurin inhibitor (tacrolimus or cyclosporine) were used.

Depending on the serologic cytomegalovirus (CMV) status, patients received CMV prophylaxis with (val-)ganciclovir over three to six months, which was initially administered intravenously (Cymevene^®^, Cheplapharm Arzneimittel GmbH, Greifswald, Germany) and then orally (Valcyte^®^, Roche Pharma AG, Grenzach-Wyhlen, Germany). In addition, trimethoprim/sulfamethoxazole (Cotrimoxazole^®^, Aliud Pharma GmbH, Laichingen, Germany) was used for Pneumocystis jirovecii prophylaxis and amphotericin B (Ampho-Moronal^®^, Dermapharm AG, Grünwald, Germany) for antimycotic prophylaxis, for three months each.

All statistical analyses were conducted using IBM SPSS Statistics 25 package (IBM, Armonk, NY, USA). Quantitative variables are expressed as mean ± standard deviation. The differences in quantitative variables between the groups were determined using Student’s *t*-test for normally distributed data and the Mann–Whitney *U* test for skewedly distributed data. Categorical variables are expressed as frequencies and percentages. Differences between the groups were calculated using Fisher’s exact test. Survival time was defined as absolute survival. Statistical significance was set at *p* < 0.05.

## 3. Results

The characteristics of the patients are presented in Table 1. A total of 24 patients (eight males, 16 females) underwent Re-SPK after previous SPK. Mean age at retransplantation was 44.4 ± 5.5 years, whereas mean age at initial transplantation was 36.0 ± 6.0 years.

The average time between primary SPK and Re-SPK was 106.5 ± 80.7 months. The mean duration of dialysis between both transplantations was 35.9 ± 31.9 months. Averagely, the initial diagnosis of type 1 diabetes mellitus was 31.7 ± 8.4 years before retransplantation.

There were no significant differences between the BMI values, lengths of hospital stay, operation times, and ischemia times of the pancreas and kidney grafts.

At the time of retransplantation, 17/24 (70.8%) patients were immunized. There was a statistically significant difference between the mean peak panel reactive antibody (PRA) values and the last measured PRA values at the time of initial transplantation and retransplantation (*p* < 0.001). Immunization after initial transplantation required the selection of organs that were as immunologically suitable as possible. Therefore, there was a statistically significant better HLA mismatch in Re-SPK than in primary SPK (*p* = 0.01). As described elsewhere, a highly immunized patient required desensitization therapy with rituximab prior to Re-SPK, followed by repeated plasmapheresis/immunoabsorption sessions and intravenous substitution of immunoglobulin [26].

Induction immunosuppressive therapy in primary SPK was predominantly performed with ATG Fresenius^®^ (Fresenius Biotech, Bad Homburg, Germany) (*p* = 0.04), while Thymoglobuline^®^ (Sanofi, Paris, France) was used more frequently in Re-SPK (*p* < 0.001). Maintenance therapy after initial transplantation consisted of steroids, cyclosporine, and azathioprine (*p* = 0.04, *p* < 0.001). Due to advancement in the pharmacological development of immunosuppressive medication, steroids, mycophenolic acid, and tacrolimus were used more frequently in cases with SPK-retransplantation (*p* < 0.001).

The donor data for the first SPK group and Re-SPK group are shown in Table 2. Most of these parameters were comparable. Significant differences were only found in the length of stay in the intensive care unit, the rate of cardiopulmonary resuscitation, and the perfusion solution used. Due to organ shortage in Germany, it has become increasingly necessary to accept organs from donors who had undergone CPR. This trend was also observed in the Re-SPK group, where the proportion of donors who had undergone CPR was significantly higher (*p* = 0.004), whereas the duration of stay in the intensive care unit was significantly longer in the first SPK group (*p* = 0.03).

The acceptance of marginal organ donors in recent years is also evident in the calculation of the pancreas donor risk index (PDRI), which was higher in the historically younger Re-SPK group. However, this difference was not statistically significant (*p* = 0.06). In 2007, the perfusion solution for visceral organ harvesting in Germany was changed from the University of Wisconsin solution (UW) to Histidine-Tryptophan-Ketoglutarate solution (HTK); thus, the proportion of HTK-perfused organs was higher in the Re-SPK group (*p* = 0.005).

### 3.1. Patient and Graft Survival, Graft Function, and Rejection Rates

The mean follow-up time after Re-SPK was 4.9 ± 4.2 years. Patient survival was 79.2% (*n* = 19) at 3 months, 75% (*n* = 18) at 1 year, and 66.7% (*n* = 16) at 5 years. The 1-year mortality rate was 25% (*n* = 6), and five (20.8%) patients died within the first 3 months after retransplantation. Causes of death were hemorrhagic shock due to arterial hemorrhage in three cases, sepsis with multiorgan failure in two cases, and unknown in one patient.

Graft survival and rejection rates are presented in Table 3. After a mean hospital stay of 48.6 ± 42.8 days, 17 patients (70.8%) were discharged with good pancreas graft function; mean serum glucose level was 93.6 ± 27.5 mg/dL with a mean HbA1c-value of 6.2 ± 0.8% at the time of discharge. Pancreas graft function was present in 16 patients (66.7%) at 1 year and in 11 patients (45.8%) at 5 years.

At discharge, 16 patients (66.7%) had good kidney graft function (creatinine, 1.67 ± 1.14 mg/dL; urea, 26.06 ± 13.93 mg/dL), and a glomerular filtration rate (calculated according to the Cockroft–Gault formula) of 61.4 ± 22.6 mL/min. Good kidney graft function was found in 15 patients (62.5%) after one year and in 13 patients (54.2%) after 5 years. The reasons for loss of graft function (besides the death of a patient) were the occurrence of postoperative complications and acute or chronic rejection.

Rejection occurred in seven patients (29.2%), five of them in the first year. Mild rejections were treated with steroid pulse and severe cellular rejection with thymoglobuline therapy.

One patient experienced an acute rejection, with evidence of donor-specific antibodies (DSA). In this case, an intensified therapy with thymoglobuline, eculizumab, and several plasmapheresis was initiated but, even after the completion of therapy, the graft failed to function.

Hospital stay of our patients was significantly longer compared to international data. However, in our department we have the possibility to perform part of the postoperative rehabilitation, which leads to a longer hospital stay. The increased length of stay is also explained by those patients with a prolonged recovery phase after postoperative complications. The median inpatient length of stay was 38 days in the first SPK group and 32.5 days in the Re-SPK group. This is comparable to the length of stay in other German transplant centers.

### 3.2. Operative Technique

Owing to existing previous operations and associated anatomical variants, Re-SPK presents a technical challenge. Furthermore, the construction of arterial vessel anastomoses is complicated by progressive vasosclerosis in the recipient and previous stent implantations. The duration of the operation and ischemic times are listed in Table 1. An overview of the surgical techniques used is presented in Table 4. Overall, the mean surgical time was 349 ± 69 min, with the mean ischemia time of the pancreas graft being 784.8 ± 161.2 min and that of the kidney graft being 858.2 ± 145.4 min.

### 3.3. Surgical Technique for Initial Transplantation

The transplantations in the first SPK group were carried out between 1994 and 2008. Since some of these were also performed in other transplant centers, complete data were not always available for all patients.

Exocrine drainage of the pancreas graft in primary SPK cases was performed using the bladder drainage technique in eight (33.3%) patients. In the modern era, small bowel drainage (duodenojejunostomy) will become increasingly established as an exocrine drainage technique, which was applied in 10 patients (41.7%). A duodenoduodenostomy was performed in one patient (4.2%).

Arterial anastomosis was performed to the right common iliac artery using an arterial Y-graft. Venous anastomosis was performed systemically in 15 patients (right common iliac vein, *n* = 12; right external iliac vein, *n* = 1; inferior caval vein, *n* = 2) and portal-venous to the superior mesenteric vein in two patients.

**Figure 1:**
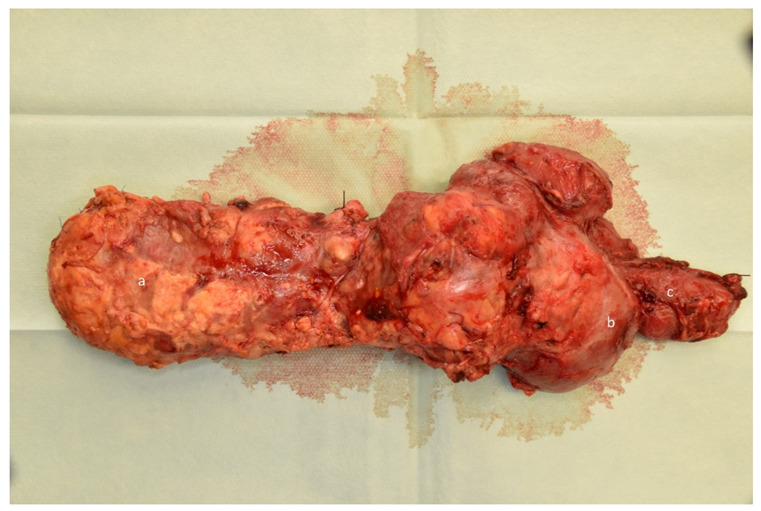
Severe graft pancreatitis. Preparation after graft pancreatectomy with severe graft pancreatitis. Exocrine drainage was performed via a duodenojejunostomy. Reconstruction of the intestinal passage was performed by jejunojejunostomy after graft pancreatectomy. a = Pancreas graft parenchyma with edematous swelling and graft pancreatitis. b = Pancreas graft duodenum with massive swelling. c = Recipient jejunum.

The vessels of the kidney graft were anastomosed to the left pelvic axis at the common iliac artery and vein in all cases. The ureteral tube was additionally anastomosed to the urinary bladder with an antireflux plastic according to the Lich-Gregoir technique using a JJ-Stent. Postoperatively, relaparotomy was necessary in 11 patients (45.8%) after the first simultaneous pancreas/kidney transplantation. In the vast majority of cases, the indications were acute bleeding or graft pancreatitis (Figure 1). Loss of function of the kidney graft was reported after a mean duration of 73 ± 72 months and that of the pancreas graft after 56.7 ± 66.3 months. This included three patients (12.5%) with an initial nonfunction.

In most cases, graft nephrectomy or pancreatectomy is justified for infectious, immunological, and anatomical reasons. In this study, graft nephrectomy was performed in 22 (91.7%) of the cases, of which fourteen (58.3%) were performed prior, seven (29.2%) during, and one (4.2%) after retransplantation. The most common indications were chronic rejection and thrombosis with an associated symptomatic graft. In one case, the indication was a postoperative recurrent urinary tract infection.

In contrast, 13 patients (54.2%) required graft pancreatectomy; In 11 of them (45.8%), this was performed prior, in one case (4.2%) during, and in one case (4.2%) after retransplantation. The most common reasons were graft rejection, bleeding, and thrombosis. In many cases, the pancreas graft shrinks to such a small size that anatomical space problems do not occur during retransplantation.

### 3.4. Surgical Technique in Re-SPK Cases

In the context of retransplantation, arterial and venous anastomoses of the pancreas graft pose a technical challenge. In most cases, the graft artery was anastomosed cranial to the right common iliac artery of the initial graft in 18 patients (75%). In three patients (12.5%), it was necessary to anastomose the pancreas graft aortally due to advanced pAVK or vascular stents inserted in the pelvic arteries. In two patients (8.3%), the right external iliac artery was chosen for anastomosis, and in another (4.2%), the arterial anastomosis was made to the stump of the Y-graft of the initial graft.

The majority of venous anastomoses were systemic venous (87.5%) (inferior caval vein, *n* = 19; right common iliac vein, *n* = 2). In three patients (12.5%), the anastomosis was portal-venous to the superior mesenteric vein. Exocrine drainage was performed in all Re-SPK cases as enteric drainage using a duodenojejunostomy in 11 (45.8%) cases and duodenoduodenostomy in 12 (50%) cases. In one patient (4.2%), duodenoileostomy was performed because of anatomical peculiarities.

Only one patient (4.2%) required graft pancreatectomy of the initial graft. In most cases, the pancreatic graft had shrunk to such a small size that no space problems were encountered during retransplantation. Vascular anastomoses of the renal graft were performed on the left pelvic axis in all patients during retransplantation. In four patients (16.7%), the external iliac artery and vein were used, and in twenty (83.3%), the common iliac artery and vein were used. In seven patients (29.2%), the old kidney graft interfered during retransplantation, so simultaneous graft nephrectomy was necessary for creation of space. In many cases, new vascular anastomoses were made cranial to the old graft. Finally, ureteroneocystostomy was performed with an antireflux plastic according to Lich-Gregoire with the application of a ureteral splint analogous to the initial transplantation.

### 3.5. Surgical Complications

Relaparotomy was indicated in 13 patients (54.2%), with the most frequent indications being acute bleeding (seven patients, 29.2%) and the occurrence of graft pancreatitis (10 patients, 41.7%). In this patient population, there was no graft thrombosis.

In one patient, relaparotomy was indicated because of abdominal pain and suspected dislocation of an inserted suprapubic catheter. One patient had relevant ureteral stenosis, and another a small bowel perforation.

Overall, in the postoperative course after 39.2 ± 35.3 days, five patients (20.8%) required graft pancreatectomy due to bleeding complications and severe graft pancreatitis. Two of these patients (8.3%) underwent simultaneous graft nephrectomy due to primary nonfunction and acute rejection with sepsis. One patient developed thrombotic occlusion of the common iliac artery due to advanced peripheral arterial occlusive disease, resulting in critical ischemia with necrosis of the lower limb. In this case, knee disarticulation was necessary.

### 3.6. Mortality Rate on Waiting List for Re-SPK

Regarding mortality rate on the waiting list for Re-SPK, we performed a subgroup analysis. A total of 17 patients were identified on the waiting list for Re-SPK after simultaneous pancreas–kidney transplantation. One patient still remains on the waiting list, whereas 16 patients (94.1%) died during the waiting period. Among these, the 1-year mortality rate was 29.4% (*n* = 5) and the 3-year mortality rate was 52.9% (*n* = 9). Causes of death were myocardial infarction (*n* = 4/25%), sepsis (*n* = 3/18.75%), cancer (*n* = 2/12.5%), hypoglycemia (*n* = 1/6.25%), and unknown (*n* = 6/37.5%).

## 4. Discussion

Simultaneous pancreas–kidney transplantation is currently the treatment of choice for patients with type 1 diabetes mellitus and end-stage renal disease due to better patient survival, prevention of diabetic sequelae, and improvement of quality of life. Overall, advancements in the development of immunosuppressive agents and surgical techniques have significantly improved graft survival (with more than 90% 1-year graft survival rate) [1,2,3,4,5,6,7,8]. Nevertheless, pancreas transplantation has a high complication rate compared to other solid organ transplantations. The most frequent reasons for graft failure are graft thrombosis, bleeding complications, the occurrence of graft pancreatitis, anastomosis insufficiency, acute or chronic rejection, and infections. Depending on the indication and clinical condition of the patient, graft pancreatectomy may be necessary and should be performed early, especially in cases of life-threatening complications [9,10,11]. For these patients with loss of graft function, retransplantation is contemplated to avoid or slow down the progression of late diabetic damage.

In the literature, there are several publications on isolated pancreas retransplantation in which very different results are presented with regard to patient and graft survival.

Overall, there is a wide variation in the 1-year survival rate of the pancreas graft, which is reported in the literature to be between 32% and 85%. The 5-year survival rate of pancreas grafts varies between 46% and 69%. However, the 1-year patient survival in these collectives was 89–96%, despite poor graft survival [12,13,14,15,16].

Patients who underwent Re-SPK after previous isolated pancreas transplantation showed an overall higher mortality rate than those in the comparison groups who only had isolated pancreas retransplantation [13]. Conversely, patients with pancreas retransplantation who had previously undergone SPK transplantation showed better kidney graft survival [17].

Loss of graft function often results from postoperative complications or rejection. Seal et al., reported lower complication rates, especially in the first three months after pancreas retransplantation, compared to initial transplantation [18]. In contrast, Humar et al. reported higher complication rates, especially the occurrence of thrombosis and bleeding [14,19,20]. In a study by Sansalone et al., graft pancreatectomy was performed in all patients (*n* = 7) in the postoperative course to avoid further complications [19]. In a study by Rudolph et al., no significant differences were found with regard to technical failure and the rate of rejection from the first and second transplants [21]. Humar et al. reported a higher rejection rate in the first year after retransplantation than in the first transplantation (55% vs 33%) [14]. This is due to higher immunization after a previous transplantation, which renders acceptance of a donor organ almost impossible in some patients for immunological reasons (e.g., the presence of unacceptable antigens). In selected cases, preoperative desensitization by means of plasmapheresis, immunoglobulin administration, and the use of thymoglobuline, rituximab, or basiliximab is possible [26].

Important criteria for appropriate recipient selection for retransplantation are immunological risk, vascular status, and existing comorbidities [27].

Careful donor selection is also considered a decisive criterion. For example, organs from donors with comorbidity, fatty or hardened pancreas grafts, or those with signs of trauma or ischemia should not be used [15,22]. In addition, a low pancreas donor risk index (PDRI) seems to be associated with better primary function [27].

For patients with Re-SPK after previous SPK, there are only a few publications in the literature with a smaller number of patients [26,27,28].

This form of retransplantation is characterized by technical and immunological challenges. Depending on the intraoperative peculiarities, a great deal of experience on the part of the surgeon is necessary. This applies to both adhesions after corresponding previous operations and creation of anastomoses in a patient with advanced vascular damage. Intraoperatively, new vascular anastomoses are usually created cranial to the initial anastomoses. In some cases, graft nephrectomy (*n* = 7) or graft pancreatectomy (*n* = 1) was required as a simultaneous procedure during Re-SPK due to space limitations. The removal of the pancreas graft was less often necessary as the graft had shrunk considerably in most. In contrast, elective graft nephrectomy was performed in most cases prior to retransplantation.

The mean ischemia time of the pancreas graft of about 13 h is comparable to the 14.5 h reported by LaMattina et al. The rate of rejection in the first year, which was 20.8% (*n* = 5) in our survey, is lower than that reported by LaMattina et al. [28].

However, contrary to what has been reported in the literature so far, our analysis shows an increased 1-year mortality rate of 25% (*n* = 6). Five of these patients (20.8%) died within three months of retransplantation due to complications. The most common complications were graft pancreatitis and hemorrhage with an associated septic course. In contrast, LaMattina et al. reported 100% patient survival at 1 and 3 years.

The 1-year survival rate of the pancreas graft in our collective was 66.7% (*n* = 16) compared to 78% in the patient collective from LaMattina and coworkers. The 1-year survival rate of the kidney graft in our study is even lower at 62.5% (*n* = 15) compared to 89% in the study by LaMattina et al. [28].

Isolated pancreas retransplantation and simultaneous pancreas–kidney retransplantation are reported as safe and effective treatment options for suitable patients in an experienced center [18,21,23,24,25,28]. The good patient survival rates in the aforementioned studies cannot be transferred to our patient collective. Despite careful and critical donor and recipient selection, there is an increased mortality rate with poor overall graft survival.

Otherwise, patients on the waiting list for Re-SPK after previous SPK also show an increased annual mortality rate. In our study, 1- and 3-year mortality rates on the waiting list were 29.4% (*n* = 5) and 52.9% (*n* = 9), respectively. Venstrom et al. also reported that type 1 diabetics with kidney failure have a significantly increased 1-year mortality rate compared to patients awaiting pancreas transplantation alone [29]. In conclusion, patients with loss of pancreas and kidney function have an increased mortality rate both after retransplantation and on the waiting list due to their increased comorbidity.

Regarding the risk profile of recipients, it should be noted that pancreas transplantation is not absolutely necessary for survival [22]. Intensified insulin therapy, the use of insulin pumps, and islet transplantation are currently alternatives to Re-SPK.

However, islet transplantation is not yet a standard therapy for diabetic patients [30]. In patients with an unstable metabolic condition and good kidney function, it can be considered as an alternative therapy option. For patients with contraindications to repeat major surgical intervention, islet transplantation would be ideal because the procedure is associated with significantly less disability. But islet transplantation after previous SPK is also limited by organ shortage and a high rate of immunized recipients. Furthermore, it should be mentioned that the regulatory situation in Germany is extremely complex. As a result, islet transplantation in Germany is not practicable in the existing structures—only one center offers this complex procedure [31]. There are only a few case reports on the outcome of islet transplantation after pancreas transplantation in the literature [32]. In particular, there are no data on islet transplantation after SPK.

Recipient selection for Re-SPK should be critically discussed in an interdisciplinary transplant conference and in the presence of experienced transplant surgeons. It is imperative to ensure that no patients with high comorbidity and an especially high cardiac risk profile are relisted. If recipients have a significantly increased risk profile (previous cardiac disease, poor vascular status, previous postoperative major complications, advanced age, high BMI), Re-SPK should be discouraged in order to avoid complications and relaparotomies. A kidney transplant alone is then more likely to be recommended for such patients. According to our experience, organs from donors with severe abdominal trauma, hematoma or fatty degeneration of the pancreas, and a PDRI > 1.3 should not be accepted for Re-SPK.

In summary, simultaneous pancreas-kidney retransplantation after a previously performed combined pancreas–kidney transplantation is a high-risk procedure, while the risk of dying on the waiting list is even higher.

## Figures and Tables

**Table 1:** Recipient characteristics.

Recipient Characteristics	First SPK	Re-SPK	*p*-Value
Gender male/female	8/16 (33.3/66.7)	8/16 (33.3/66.7)	ns
Age (years)	36.0 ± 6.0	44.4 ± 5.5	<0.001
Time to Re-SPK (months)		106.5 ± 80.7	
Weight (kg)	65.7 ± 10.6	70.1 ± 11.3	ns
Height (cm)	173.8 ± 10.1	173.8 ± 10.1	ns
BMI (kg/m^2^)	22.0 ± 2.3	23.2 ± 2.9	ns
Diabetes duration (years)	22.5 ± 6.4	31.7 ± 8.4	<0.001
Dialysis duration (months)	27.2 ± 28.4	35.9 ± 31.9 *	ns
PRA highest (%)	3.9 ± 8.6	41.6 ± 39.1	<0.001
PRA last (%)	1.2 ± 4.9	29.1 ± 36.4	<0.001
HLA mismatch			
HLA-A	1.3 ± 0.6	1.0 ± 0.8	ns
HLA-B	1.8 ± 0.4	1.08 ± 0.8	<0.001
HLA-DR	1.4 ± 0.7	1.33 ± 0.7	ns
HLA Total	4.5 ± 1.0	3.42 ± 1.6	0.01
CMV serology			
R+ D−	6 (25)	10 (41.7)	
R+ D+	6 (25)	5 (20.8)	
R− D+	6 (25)	6 (25)	
R− D−	6 (25)	3 (12.5)	
Operation			
Operation time (min)	329.4 ± 113	349 ± 69	ns
Pancreas cold ischemic time (min)	788.3 ± 221.3	784.8 ± 161.2	ns
Kidney cold ischemic time (min)	824.3 ± 226	858.2 ± 145.4	ns
Induction therapy			
ATG Fresenius	16 (66.7)	8 (29.2)	0.04
ATG Tecelac	0	1 (4.2)	ns
Thymoglobuline	2 (8.3)	14 (58.3)	<0.001
Daclizumab	0	1 (4.2)	ns
Immunosuppressive regimen			
Steroid	24 (100)	24 (100)	ns
MPA	9 (37.5)	24 (100)	<0.001
Azathioprine	9 (37.5)	0 (0)	<0.001
Tacrolimus	8 (33.3)	23 (95.8)	<0.001
Cyclosporine	10 (41.7)	1 (4.2)	0.004

Values are presented as mean ± SD or *n* (% of group). Simultaneous pancreas–kidney transplantation (SPK), simultaneous pancreas–kidney retransplantation (Re-SPK), not significant (ns), body mass index (BMI), panel reactive antibodies (PRA), human leukocyte antigen (HLA), cytomegalovirus (CMV), and mycophenolic acid (MPA). * Dialysis duration after first SPK.

**Table 2:** Donor characteristics.

Donor Characteristics	First SPK	Re-SPK	*p*-Value
Gender (male/female)	14/10 (58.3/41.7)	10/14 (41.7/58.3)	ns
Age (years)	27.1 ± 12.3	29.5 ± 12.9	ns
BMI (kg/m^2^)	22.9 ± 2.7	23.3 ± 3.7	ns
Height (cm)	175.5 ± 8.4	168.8 ± 11.3	ns
Weight (kg)	69.8 ± 12.1	67.3 ± 14.2	ns
Serum creatinine (mg/dL)	0.92 ± 0.23	0.95 ± 0.5	ns
Serum urea (mg/dL)	34.6 ± 17.2	34.1 ± 30.3	ns
GFR (mL/min) (Cockroft–Gault formula)	117.7 ± 26.4	113.7 ± 49.6	ns
Serum amylase (U/l)	177.8 ± 156.5	109.9 ± 109.9	ns
ICU stay (days)	4.5 ± 4.2	2.71 ± 1.7	0.03
Hypertension	0 (0)	3 (12.5)	ns
Smoking	1 (4.2)	5 (20.8)	ns
Alcohol consumption	1 (4.2)	5 (20.8)	ns
Resuscitation	0 (0)	7 (29.2)	0.004
Perfusion solution (UW/HTK/unknown)	19/4/1 (79.2/16.7/4.1)	9/14/1 (37.5/58.3/4.1)	0.005
PDRI	1.09 ± 0.23	1.28 ± 0.4	0.06

Values are presented as mean ± SD or *n* (% of group). Simultaneous pancreas–kidney transplantation (SPK), simultaneous pancreas–kidney retransplantation (Re-SPK), not significant (ns), body mass index (BMI), glomerular filtration rate (GFR), intensive care unit (ICU), University of Wisconsin solution (UW), histidine-tryptophan-ketoglutarate (HTK), pancreas donor risk index (PDRI).

**Table 3:** Graft survival and rejection rates after Re-SPK.

	First SPK	Re-SPK	*p*-Value
Hospital stay (days)	47 ± 23.4	48.6 ± 42.8	ns
Pancreas graft function			
At discharge from hospital	12 (50)	17 (70.8)	ns
Serum glucose (mg/dL)	108.5 (*n* = 12)	93.6 ± 27.5	0.05
HbA1c (%)	6 ± 0.2 (*n* = 10)	6.2 ± 0.8	ns
1-year graft survival	8 (33.3)	16 (66.7)	0.04
5-year graft survival	6 (25)	11 (45.8)	ns
Kidney graft function			
At discharge from hospital	18 (75)	16 (66.7)	ns
Serum creatinine (mg/dL)	1.49 ± 0.5 (*n* = 17)	1.67 ± 1.1	ns
Serum urea (mg/dL)	17.1 ± 4.4(*n* = 17)	26.06 ± 13.9	0.02
GFR (mL/min)	71.3 ± 39.9	61.4 ± 22.6	ns
1-year graft survival	12 (50)	15 (62.5)	ns
5-year graft survival	11 (45.8)	13 (54.2)	ns
Rejection rate			
1 year rejection rate	5 (20.8)	5 (20.8)	ns
5 year rejection rate	8 (33.3)	7 (29.2)	ns
Rejection grade			ns
Borderline	0 (0)	1 (14.3)	
Banff grade I	5 (62.5)	4 (57.1)	
Banff grade II	2 (25)	1 (14.3)	
Humoral	1 (12.5)	1 (14.3)	

Values are presented as mean ± SD or *n* (% of group). Simultaneous pancreas-kidney transplantation (SPK), simultaneous pancreas–kidney retransplantation (Re-SPK), not significant (ns), glomerular filtration rates (GFR).

**Table 4:** Operation technique.

Operation Technique	First SPK	Re-SPK
Pancreatic anastomoses		
Exocrine: BD/DJ/DD/DI	8/10/1/0 (33.3/41.7/4.2/0)	0/11/12/1 (0/45.8/50/4.2)
Arterial: RCIA/REIA/SMA	15/0/2 (62.5/0/8.3)	18/2/0 (75/8.3/0)
Arterial: y-graft stump/aorta	0/0 (0/0)	1/3 (4.2/12.5)
Venous: RCIV/REIV/IVC/SMV	12/1/2/2 (50/4.2/8.3/8.3)	2/0/19/3 (8.3/0/79.2/12.5)
Kidney anastomoses		
Arterial: LCIA/LEIA	11/6 (45.8/25)	20/4 (83.3/16.7)
Venous: LCIV/LEIV	11/6 (45.8/25)	20/4 (83.3/16.7)
Relaparotomy rate	11 (45.8)	13 (54.2)
Bleeding	4 (16.7)	7 (29.2)
Graft pancreatitis	6 (25)	10 (41.7)
Graft thrombosis	4 (16.7)	0 (0)
Other	0 (0)	3 (12.5)
Graft nephrectomy	22 (91.7)	2 (8.3)
Prior to Re-SPK	14 (58.3)	0 (0)
At Re-SPK	7 (29.2)	0 (0)
Post Re-SPK	1 (4.2)	2 (8.3)
Loss of function KTx (months)	73 ± 72	31.3 ± 51.4
Time to graft nephrectomy (d)	2550.2 ± 2336.2	39.2 ± 35.3
Graft pancreatectomy	13 (54.2)	5 (20.8)
Prior to Re-SPK	11 (45.8)	0 (0)
At Re-SPK	1 (4.2)	0 (0)
Post Re-SPK	1 (4.2)	5 (20.8)
Loss of function PTx (months)	56.7 ± 66.3	31.1 ± 51.1
Time to graft pancreatectomy (d)	242.8 ± 509.8	39.2 ± 35.3

Values are presented as mean ± SD or *n* (% of group). Simultaneous pancreas–kidney transplantation (SPK), simultaneous pancreas–kidney retransplantation (Re-SPK), bladder drainage (BD), duodenojejunostomy (DJ), duodenoduodenostomy (DD), duodenoileostomy (DI), right common iliac artery (RCIA), right external iliac artery (REIA), superior mesenteric artery (SMA), right common iliac vein (RCIV), right external iliac vein (REIV), inferior vena cava (IVC), superior mesenteric vein (SMV), left common iliac artery (LCIA), left external iliac artery (LEIA), left common iliac vein (LCIV), left external iliac vein (LEIV), kidney graft (KTx), and pancreas graft (PTx).

## Data Availability

Data sharing is not applicable to this article.
